# Lysergic Acid Amide (LSA), an LSD Analog: Systematic Review of Pharmacological Effects, Adverse Outcomes, and Therapeutic Potentials

**DOI:** 10.3390/pharmacy13040098

**Published:** 2025-07-21

**Authors:** Paula S. C. C. Castro, Kae Leopoldo, Maria Olivia Pozzolo Pedro, Juliana Takitane, Henrique Silva Bombana, André Brooking Negrão, Jaqueline R. Scholz, João Maurício Castaldelli-Maia

**Affiliations:** 1 Department of Neuroscience, Medical School, FMABC University Center, Santo André 09060-870, SP, Brazil; paula.castro@aluno.fmabc.net; 2 Department of Psychiatry, Medical School, University of São Paulo, São Paulo 05403-903, SP, Brazil; kae.leopoldo@usp.br (K.L.); maria.oliviapozzolo@usp.br (M.O.P.P.); 3 Instituto Perdizes (IPER), Hospital das Clínicas HCFMUSP, Medical School, University of São Paulo, São Paulo 05021-001, SP, Brazil; abnegrao@usp.br; 4 Department of Experimental Psychology, Psychology Institute, University of São Paulo, São Paulo 05508-030, SP, Brazil; 5 LIM/40—Laboratory of Genetics and Forensic Toxicology, Medical School, University of São Paulo, São Paulo 05405-150, SP, Brazil; julianatakitane@gmail.com; 6 School of Pharmaceutical Sciences, University of Sao Paulo, São Paulo 05508-000, SP, Brazil; hbombana@usp.br; 7 Prevention Program, Heart Institute (InCor), Hospital das Clínicas (HCFMUSP), Medical School, University of São Paulo, São Paulo 05403-900, SP, Brazil; jaqueline@incor.usp.br

**Keywords:** lysergic acid amide (LSA), legal highs, psychotropic substances, cluster headaches, systematic review

## Abstract

**Objective**: To systematically review the scientific literature on lysergic acid amide (LSA), focusing on its physical, neurobiological, and social effects, as well as its potential risks and therapeutic uses. **Methods**: A systematic review was conducted across PubMed, Google Scholar, and Web of Science up to December 2023, using keywords such as “ergine,” “lysergic acid amide,” and “legal high.” Studies were included if they reported original human data on the physical, neurobiological, psychological, or social effects of LSA; seventeen studies were included. Animal studies, in vitro research, and non-original articles were excluded. Two independent reviewers screened and selected the studies, with a third resolving discrepancies. Data were extracted using a standardized form. The review followed PRISMA guidelines and was prospectively registered on the Open Science Framework. **Results***:* LSA is primarily consumed through preparations made from the seeds of Convolvulaceae plants. Reported effects include euphoria, hallucinations, nausea, and anxiety. Severe adverse outcomes, such as psychosis, hypertension, and hospitalization, have also been documented. Some evidence suggests its potential therapeutic application for cluster headaches. However, variability in dosing and misinformation on digital platforms heighten the risks associated with LSA use. **Conclusions***:* LSA poses significant health risks, exacerbated by online misinformation and variability in its effects, and a lack of scientific studies. Further research is essential to clarify its pharmacological profile, establish guidelines for safe use, and raise public awareness about its dangers.

## 1. Introduction

Psychedelics are a class of substances used for recreational, ritualistic [1], and research purposes [2]. These compounds induce altered states of consciousness with psychomimetic effects that depend significantly on the user and the environment [3]. Chemically, psychedelics can be divided into three main categories: indoelamines (e.g., N,N-dimethyltryptamine, phenethylamines (e.g., mescaline), and ergolines (e.g., ergolines such as lysergic acid diethylamide [LSD] and related compounds) [1]. Hallucinogens generally exert their effects by stimulating 5-HT(2A) receptors, particularly those on neocortical pyramidal cells, which also increase cortical glutamate levels. They have a low affinity for receptors in the autonomic nervous system, leading to low physiological toxicity. This is not only due to their receptor affinity but also their high pharmacological potency, as in the case of LSD, which is active in doses as low as 50–100 µg. They also have limited effects on dopaminergic receptors in the mesolimbic area, which may explain their low potential for dependence [1,4]. Although hallucinogens do not typically cause overdose or dependence, certain health issues can arise from their use. For example, hallucinogen-induced perception disorder involves persistent changes in perception, including hallucinations reminiscent of intoxication, even when sober, causing significant clinical distress. Additionally, psychedelics can trigger bipolar, depressive, or psychotic disorders induced by hallucinogens [5,6,7]. Despite these risks, psychedelics are increasingly being explored in the field of mental health, with studies investigating their potential for treating anxiety and other psychiatric disorders [8].

Psychedelics are frequently consumed for recreational purposes. However, most of these substances are illegal and difficult to obtain, creating a demand for legal alternatives, known as “legal highs” [9]. Lysergic acid amide (LSA) is one such psychoactive substance, sought after for its effects similar to LSD [10]. However, the negative effects of LSA on human physiology remain poorly understood by medical science.

LSD remains one of the most commonly used hallucinogens for recreational purposes; it is rapidly absorbed, has a half-life of approximately three hours, and undergoes hepatic metabolism [11]. While no fatalities from overdose have been reported, a case study documented the effects of high-dose intranasal LSD ingestion, mistakenly taken as cocaine. Patients experienced coma, hyperthermia, vomiting, mild gastric bleeding, and respiratory issues, but all recovered without fatalities [12]. LSD acts as a partial agonist of 5HT1 and 5HT2 receptors. Evidence also suggests that LSD interacts with dopaminergic systems, acting as both an agonist and antagonist at D1 and D2 receptors, although its influence on the drug’s psychoactive effects remains unclear [13].

**Figure 1:**
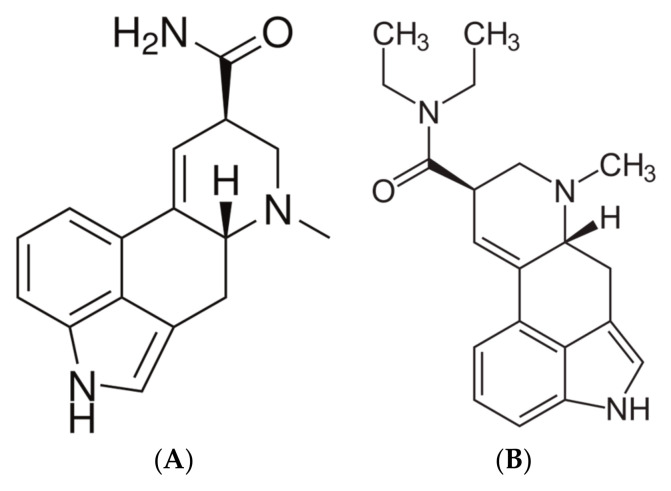
LSA (**A**) and LSD (**B**) chemical structures.

Novel Psychoactive Substances (NPSs) are synthetic psychoactive compounds that are not subject to international control under the “United Nations 1961 Single Convention on Narcotic Drugs or the 1971 Convention on Psychotropic Substance” [14,15]. NPS includes substances in the form of plants, herbal mixtures, or synthetic preparations that mimic the effects of illicit drugs. They can be commercialized as legal alternatives to illicit substances like LSD, cannabis, or amphetamines, and in that instance are known as “legal highs”. Their pharmacology and toxicology are poorly defined due to limited scientific data, raising concerns about their safety [16]. Ergine, also known as LSA, is an ergot alkaloid structurally like LSD (Figure 1) but with distinct and partially unexplored pharmacological effects. It is primarily found in the seeds of plants from the Convolvulaceae family, particularly *Argyreia nervosa* (AN), which has been used historically in rituals and traditional medicine by indigenous populations [16]. In modern contexts, LSA is consumed as a “legal high” due to its LSD-like effects and accessibility. It can be purchased online or from gardening stores. However, online forums and digital platforms often provide misleading information about extraction and ingestion methods, increasing the risk of poisoning from harmful seed components or ineffective extraction processes [17]. Due to the lack of scientific information about the physical and psychological effects of LSA in humans, this study aims to systematically review the scientific literature on LSA, focusing on its physical, neurobiological, and social effects, as well as its potential risks and therapeutic uses.

To the best of our knowledge, this is the first systematic review to synthesize original human data on the use of lysergic acid amide (LSA), integrating its pharmacological effects, toxicity profile, motivations for use, and potential therapeutic applications—particularly in the context of cluster headaches. The prior literature has addressed these aspects separately or in non-systematic formats, limiting the field’s ability to consolidate evidence.

## 2. Materials and Methods

### 2.1. Eligibility Criteria and Search Strategy

This review included articles reporting original data on the physical effects, neurobiological aspects, epidemiology, bodily symptoms, and social and cultural factors related to LSA consumption in humans. Eligible articles were published in English, Portuguese, Spanish, Italian, or French, reflecting the linguistic proficiency of the research team. Studies involving animals, in vitro experiments, botanical research, and/or non-original data were excluded. The systematic review protocol was registered on the Open Science Framework (OSF) (https://osf.io/ervkm/, accessed on 16 July 2025).

Relevant articles were identified by systematically searching PubMed, Google Scholar, and Web of Science databases up to December 2023. While databases such as EMBASE and PsycINFO contain valuable pharmacological and toxicological records, we selected PubMed, Web of Science, and Google Scholar for their broad access to peer-reviewed biomedical literature and gray literature. We acknowledge that the inclusion of additional databases may further enhance coverage.

The search was conducted using the following keywords: (ergine OR “d-lysergic acid amide” OR “d-lysergamide” OR “lysergic acid amide”) OR (“legal high” AND “LSA”). The search adhered to the Preferred Reporting Items for Systematic Reviews (PRISMA) guidelines [18] to ensure transparency and reproducibility. The prefix “d-“ refers to the dextrorotatory (dextro) isomer of lysergic acid derivatives, which is the stereoisomer associated with psychoactive and pharmacological activity in humans. This nomenclature distinguishes it from the levorotatory (l-) form, which is pharmacologically inactive.

In June 2025, we re-ran the PubMed search using an expanded strategy that included both MeSH terms and relevant synonyms to ensure completeness. The terms used were: “Lysergic Acid Amide” [MeSH Terms], “lysergic acid amide,” “d-lysergic acid amide,” “d-lysergamide,” “ergine,” “LA-111,” “legal high,” and “novel psychoactive substances.” Despite the expanded search, no additional eligible studies were identified.

### 2.2. Study Selection and Data Extraction

Two independent reviewers (the first and second authors) screened the titles and abstracts of all retrieved articles. Full-text reviews were conducted for articles meeting the inclusion criteria, and any disagreements were resolved by discussion with a third reviewer. Duplicate studies were removed. Articles were included if they reported original human data on LSA effects, regardless of study design. Studies involving animal models, in vitro research, or lacking original data were excluded. Data extraction was performed independently by two reviewers using a standardized form, collecting information on author(s), year of publication, sample characteristics, study design, and main findings. Additional details on intoxication effects were categorized into cardiovascular, gastrointestinal, neurological, psychological, and other symptoms.

## 3. Results

**Figure 2:**
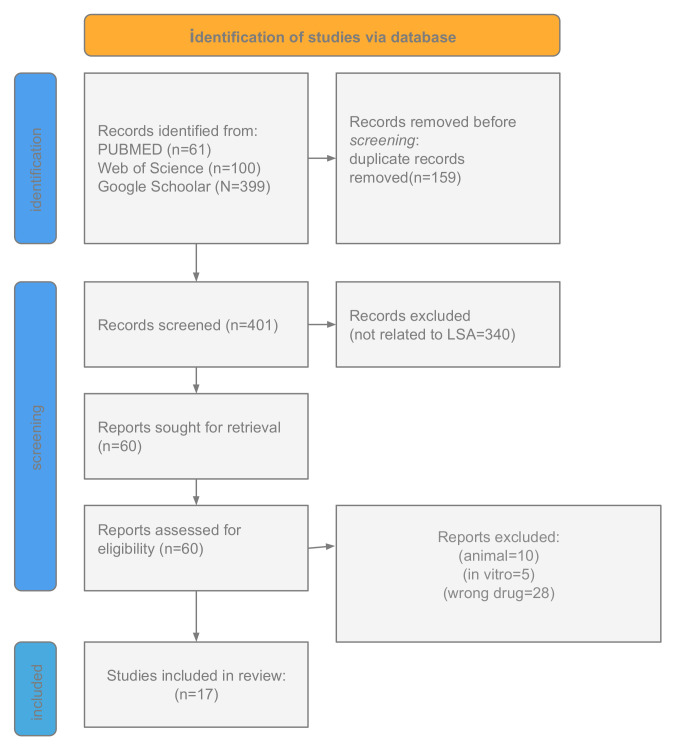
PRISMA flow diagram.

Seventeen studies investigating the availability, pharmacological effects, and adverse outcomes of LSA in humans were included in this review, as summarized in Figure 2. Table 1 presents the main findings of the studies included in this review.

### 3.1. Sources and Concentrations of LSA

LSA was primarily sourced from seeds of *Argyreia nervosa* (AN), with additional reports of *Ipomoea tricolor* (IT), *Ipomoea violacea* (IV), *Ipomea Volubilis*, *Turbina* (*Rivea*) *corymbosa*, Morning Glory, Hawaiian Baby Woodrose, herbal preparations called Druids fantasy, low potency flash inspiration, and happy caps; there was also one study with a synthetic preparation [17,19,20,21,22,23,24,25,26,27,28,29,33]. Analytical studies demonstrated that LSA is the most abundant ergot alkaloid in these seeds, though its concentration varied significantly across batches, rendering the dose unpredictable [17,19,20,21,22,23,24,25,26,27,28,29,33]. Synthetic preparations marketed as containing LSA were often found to lack the compound entirely [25]. There were other ergot alkaloids in the batches analyzed, such as iso-LSA, ergometrine, and ergometrinine [19,32]. When available, information on the ingested dose or seed quantity was extracted. For example, Paulke et al. (2012) reported a dose of 5.88 mg/kg body weight of an *Argyreia* nervosa seed preparation [28], while other reports mentioned the ingestion of 3 to 10 seeds. LSA was also quantified in biological matrices, such as blood and urine, using LC-MS/MS and other methods [28,33], although correlation with symptom severity varied due to inconsistent timing of sample collection and co-ingestants.

These findings underscore the lack of standardization in LSA preparations, complicating both recreational use and clinical investigation. Unlike LSD, whose dose–response curve is well established, LSA remains unpredictable, posing challenges for any potential therapeutic application.

### 3.2. Patient Sample and Intoxication Effects

A total of 219 patients were analyzed across studies, with 189 characterized regarding intoxication effects [23,24,25,26,27,28,29]. The most reported symptoms included cardiovascular issues (e.g., tachycardia, hypertension), gastrointestinal complaints (e.g., nausea, vomiting, abdominal pain), and psychological disturbances (e.g., paranoid thoughts, hallucinations). Approximately 27 patients (12.3%) required medical attention after LSA use, with no overdoses recorded [23,24,27]. However, severe cases included a suicide following agitation and psychosis [27] and a case of posterior reversible encephalopathy syndrome (PRES) likely induced by LSA [23]. Neurological symptoms such as seizures, tremors, and psychosis-like states were also observed. The relatively high proportion of patients requiring medical attention (12.3%)—despite the low lethality—suggests that while LSA may not be fatal, its safety profile is far from benign. The presence of severe neuropsychiatric outcomes such as PRES or suicide-related agitation warrants particular caution.

### 3.3. Therapeutic Use of LSA

Three studies investigated LSA’s potential therapeutic application in headache disorders, analyzing 120 patients recruited primarily from online forums [20,21] and one case report [23]. Approximately 20% of patients reported effectiveness in preventing cluster headaches [18], while the case report documented symptom relief for up to two weeks [22]. However, these studies also highlighted the need for controlled dosing and further clinical trials due to the variability in LSA’s effects. Although preliminary findings suggest possible benefits in headache disorders, the absence of dosage control and reliance on self-reported outcomes make it impossible to draw robust clinical conclusions.

### 3.4. Motivations for Use

Studies exploring the motivations for LSA use revealed diverse reasons, including recreational purposes, curiosity, cost-effectiveness compared to LSD, and perceived legality [30,31,32]. Some users sought LSA for cluster headache management or as an alternative when other substances were unavailable [20,21,22]. Online forums and anecdotal recommendations were significant drivers of use, despite frequent misinformation about the substance’s safety and effects [17].

### 3.5. Adverse Effects by Source

Table 2 details intoxication effects based on LSA sources. Preparations derived from AN seeds were associated with cardiovascular and gastrointestinal symptoms, mydriasis, and psychosis-like states. Synthetic preparations, which were identified as possibly not having LSA, caused tachycardia and other symptoms unrelated to LSA due to adulteration with different compounds. Hallucinations, agitation, and disorientation were commonly observed across multiple sources.

## 4. Discussion

The predominance of *Argyreia nervosa* (AN) as the main natural source of LSA raises concerns regarding safety and reproducibility. Unlike pharmacologically regulated substances, these seeds exhibit considerable variability in alkaloid content, including other compounds such as iso-LSA, ergometrine, and chanoclavine I and II, which do not share the same intoxicating effects as LSA [19,26,28,33]. This lack of chemical standardization severely limits the possibility of controlled use, whether recreational or therapeutic. Furthermore, the widespread availability of these seeds through online platforms—where the information provided is often incomplete or misleading—amplifies the risk of adverse outcomes.

The heterogeneity in motivations for LSA use reflects both its perceived therapeutic promise and its appeal as a legal alternative to more restricted psychedelics [20,21,23,31,32]. However, the pattern of intoxication effects reported—ranging from nausea, fatigue, increased blood pressure, tachycardia, hallucinations, gastrointestinal disturbances, to psychomotor agitation and persecutory thoughts [22,23,25,27,28,30]—suggests a poor risk–benefit balance in unsupervised settings. The inability to establish a dose–response relationship further complicates the clinical translation of user experiences and highlights a pressing need for laboratory-controlled trials to determine thresholds for safety and efficacy.

LSA’s distinct pharmacological profile compared to LSD—marked by stronger autonomic effects and lower serotonergic receptor affinity—may contribute to its reduced psychoactive potency, but also to an unfavorable side effect profile. While this theoretically decreases its abuse potential, it also undermines its therapeutic promise unless its pharmacodynamics are more precisely characterized. An in vitro study not included in this review suggests that LSA has a lower affinity for 5HT1 and 5HT2 receptors compared to LSD [34], which may partly explain its diminished psychotomimetic activity. However, the pharmacological actions of LSA remain insufficiently understood, and its profile raises significant concerns. The inconsistency in reported doses and variability in LSA concentration across plant batches complicate the interpretation of therapeutic versus toxic thresholds. Nevertheless, studies that measured serum or urinary concentrations following ingestion [28,33] provide a foundation for future pharmacokinetic analyses, which are crucial for safe clinical application.

Preliminary reports suggesting LSA’s utility in managing cluster headaches are intriguing, particularly given the debilitating nature of CH [35] and the limited options for prophylaxis [20,21,23]. However, these data stem from observational and anecdotal sources—often lacking dose control, randomization, or psychiatric screening. The placebo effect cannot be ruled out, especially considering the high expectation bias in online user communities. Therefore, while this finding justifies further investigation, it should not yet be interpreted as evidence of efficacy [22].

In light of these findings, public health strategies must urgently address the dissemination of accurate information about LSA. Harm reduction approaches should prioritize standardized labeling for plant-based preparations, public warnings about variable potency and psychosis risk, and stronger oversight of online markets [23,27]. In parallel, the development of clinical research protocols—anchored in ethical sourcing and respect for traditional knowledge—may enable a more responsible exploration of LSA’s therapeutic potential [19,20,21,22].

Despite its similarities to LSD, LSA appears to have a lower potential for abuse due to its more pronounced autonomic effects, such as gastrointestinal disturbances, and its relatively mild psychotomimetic effects. Additionally, the variability in LSA concentrations across sources presents a significant barrier to its widespread use for recreational purposes. However, its easy availability and reports of severe adverse effects indicate a need for greater regulatory oversight and public awareness.

Beyond the pharmacological and public health concerns associated with LSA, ethical and legal challenges also arise regarding its potential therapeutic development. Many of the plants from which LSA is derived—such as *Argyreia nervosa*—have a long history of traditional use in ritualistic and medicinal practices by Indigenous populations, particularly in Asia and Oceania. This ancestral use constitutes what is internationally recognized as traditional knowledge, which may pose significant legal and ethical barriers to the patenting of compounds extracted from these plants. According to the Convention on Biological Diversity (CBD) and related frameworks, the use of traditional knowledge requires prior informed consent and equitable benefit-sharing with the communities that hold such knowledge. These considerations highlight the need for interdisciplinary approaches that respect the rights of traditional communities when exploring the scientific and commercial potential of substances like LSA.

### 4.1. Limitations of the Review

This review has several limitations. Many of the included studies had small sample sizes and heterogeneous methodologies, making direct comparisons difficult. Specifically, four case reports [12,13,19,21], five studies with limited chemical analyses [19,26,28,33], and three observational studies on LSA efficacy for CH that relied on self-reported outcomes [20,21,22] contribute to potential biases. Additionally, some studies, such as those investigating motivations for use [31,32], were not directly focused on LSA, reducing their relevance to the review’s objectives.

Moreover, the absence of studies analyzing the long-term effects of LSA’s pharmacological properties, safety profile, and potential therapeutic applications limits the conclusions that can be drawn. In addition, one limitation of this review is the exclusion of databases such as EMBASE and PsycINFO, which may have contained additional relevant studies. Future reviews should consider expanding database coverage to ensure broader retrieval.

### 4.2. Future Research Directions

Further research is urgently needed to elucidate the pharmacological properties, toxicology, and therapeutic potential of LSA. Controlled clinical trials should explore its efficacy in CH and other conditions, while studies on its mechanism of action could provide insights into its effects compared to other psychedelics. Finally, public health initiatives should focus on regulating its sale and educating users to minimize harm.

## 5. Conclusions

LSA appears to have a lower potential for abuse compared to LSD, primarily due to its milder psychotomimetic effects and more pronounced autonomic side effects. Although its therapeutic potential in CH management is promising, current evidence remains insufficient to support widespread medical use. Furthermore, the unpredictability of its effect and lack of regulation pose significant health risks. Therefore, further research and public health interventions are essential to better understand its risks, benefits, and long-term impact.

## Figures and Tables

**Table 1:** The main findings of the studies included in this systematic review.

Author & Year (Ref)	Sample	Study Design	Main Results
Paulke et al. (2014) [19]	AN seeds (*Argyreia nervosa*) in herbal preparation (Druids Fantasy—DF)	Analytical chemistry study	LSA concentration in seeds is highly variable; other ergot alkaloids are present.
Schindler et al. (2015) [20]	A total of 108 patients from the cluster headache (CH) clinic websites	Observational research	LSA used as a preventive medication for CH; 20% found it effective.
Lorenzo et al. (2015) [21]	A total of 12 patients from the CH websites	Observational research	Nine patients reported the effectiveness of LSA for CH relief; three reported no effect.
Johnson & Black (2020) [22]	One woman who ingested Hawaiian Baby Woodrose (HBW) seeds	Case report	Reported relief from CH and mental health symptoms for two weeks.
Legriel et al. (2008) [23]	One man who ingested LSA	Case report	Seizure and posterior reversible encephalopathy syndrome (PRES) attributed to LSA use; 9-day hospitalization.
Forrester (2019) [24]	A total of 29 patients from Texas poison centers who ingested LSA	Observational research	Symptoms included tachycardia, hypertension, nausea, hallucinations, and lethargy. Twenty patients needed hospitalization or further medical care.
Bjornstad (2009) [25]	Seven patients (two bioanalytically confirmed)	Case series study	Synthetic samples often lacked LSA; seeds caused vomiting, leukocytosis, and tachycardia.
Kremer et al. (2011) [26]	Four different batches of AN	Pharmacokinetic study	LSA concentration in seeds varied unpredictably.
Klinke et al. (2009) [27]	Two patients who ingested HBW seeds	Case report	One patient experienced agitation and died by suicide after LSA use; LSA was detected in the blood.
Paulke et al. (2012) [28]	Four subjects who ingested AN seeds	Quantitative analytical validation study	Study terminated due to side effects (e.g., nausea, tremor, hypertension, psychosis-like states).
Ponté & Lapeyere-Mestre (2017) [29]	Four patients who ingested *Ipomoea volubilis* or Happy Caps (HC)	Case report	Symptoms included tachycardia, hallucinations, psychomotor agitation, and unpleasant feelings.
Juszczack & Swiergel (2013) [30]	A total of 27 patients who ingested LSA	Observational research	Motivation included curiosity and cost-effectiveness. 25% had negative experiences with symptoms such as persecutory thoughts.
Schmidt et al. (2010) [17]	Analysis of legal highs sold on UK websites	Descriptive cross-sectional study	LSA preparations marketed as hallucinogens; some lacked side effects reported by users.
Wiecko et al. (2016) [31]	A total of 26 patients interviewed about motivations for legal high use	Qualitative research study	Motivations included novelty, availability, and perceived safety.
Van Hout et al. (2011) [32]	A total of 32 adults (ages 18–33) who used legal highs	Ethnopharmacological study	Motivations included online recommendations, rarity of fatalities, and prior positive experiences.
Paulke et al. (2014) [19]	AN seeds from Madagascar (high potency) and Holrose (low potency)	Analytical chemistry investigation	LSA and other alkaloids found in all seeds; concentrations were highly variable.
Bjornstad et al. (2009) [33]	Urine of patients who ingested 10 plant-derived substances	Analytical method development study	LSA detected in urine using multi-component LC-MS/MS analysis.

CH = cluster headache, PRES = posterior reversible encephalopathy syndrome.

**Table 2:** Intoxication effects by different sources of LSA.

Author & Year (Ref)	Hospitalization	Source of LSA	Cardiovascular Effects	Gastrointestinal Effects	Neurological Effects	Psychological Effects	Other Effects
Legriel et al. (2008) [23]	1	LSA	None	None	Seizure and PRES	None	None
Klinke et al. (2009) [27]	1	Hawaiian Baby Woodrose (HBW)	None	None	None	Agitation, suicide (3 h post-use)	None
Kremer et al. (2011) [26]	0	AN seeds (1.73 μg/mg)	None	Nausea, vomiting	Tremor, weakness, fatigue	Paranoid thoughts, delusions, psychosis-like state	None
Bjornstad (2009) [25]	0	LSA/synthetic LSA	Tachycardia (synthetic)	Vomiting (seeds)	None	Hallucinations	Mydriasis, leukocytosis (seeds)
Forrester (2019) [24]	25	Beads, pieces, or pills of AN	Hypertension, tachycardia	Abdominal pain, hematemesis, nausea, vomiting	Agitation, ataxia, confusion, lethargy	Hallucinations, delusions	Muscle weakness, blurred vision, urinary retention
Paulke et al. (2012) [28]	0	AN seed preparation (5.88 mg/kg body weight)	Blood pressure elevation	Nausea	Weakness, fatigue, tremor	Psychosis-like state	None
Juszczack & Swiergel (2013) [30]	0	AN, *Ipomoea tricolor* (IT), *Ipomoea violacea* (IV), *Ipomoea purpurea* (IP), Druids Fantasy (DF)	None	Nausea, vomiting (varied sources)	None	Visual/auditory distortions, positive and negative emotions	Mydriasis (all sources)
Ponté & Lapeyere-Mestre (2017) [29]	0	*Ipomoea volubilis* (IV) and Happy Caps (HC)	Tachycardia	None	Disorientation, psychomotor agitation	Intense unpleasant feelings, hallucinations	Mydriasis

## Data Availability

All the data generated during the study is presented in the manuscript.
